# Identification of DNA Methyltransferase/Demethylase Genes and 5-Azacytidine’s Impact on β-Elemene and Methylation in *Curcuma wenyujin*

**DOI:** 10.3390/biology14121739

**Published:** 2025-12-04

**Authors:** Xiu Yin, Jiawei Ma, Zhenlu Shen, Qian Wang, Mengdie Xu, Tianyuan Hu, Qiuhui Wei, Xiaopu Yin, Xiaoxia Ma

**Affiliations:** 1 School of Pharmacy, Hangzhou Normal University, Hangzhou 311121, China; yinxiu777@163.com (X.Y.);; 2 Zhejiang Provincial Key Laboratory of Anti-Cancer Chinese Medicines and Natural Medicines, Hangzhou Normal University, Hangzhou 311121, China

**Keywords:** DNA methylation, *C. wenyujin*, CwC5-MTases, CwdMTase, β-elemene

## Abstract

*Curcuma wenyujin* (*C. wenyujin*) is a Dao-di geoherb. It depends on specific ecological conditions. DNA methylation (5mC) mediates environmental stress responses, regulating both growth and bioactive compound synthesis. This implies epigenetic control of secondary metabolism in *C. wenyujin*. However, its DNA methylation patterns remain uncharacterized. In this study, we identified five CwC5-MTases and three CwdMTases based on the transcriptome of *C. wenyujin*. They were responsible for DNA methylation and demethylation, respectively. Structural and integrated phylogenetic analysis classified the five CwC5-MTases into four subfamilies: CwMET, CwCMT, CwDRM, CwDNMT. The three CwdMTases were grouped into the ROS subfamily. Both CwC5-MTases and CwdMTases exhibited the closest evolutionary relationship to their homologs in monocots. Treatment of *C. wenyujin* seedlings with the DNA methyltransferase inhibitor 5-azacytidine (5-Az) enhanced terpenoid biosynthesis. QPCR analysis demonstrated that this treatment significantly upregulated key biosynthetic genes, with the exception of *CwDXS*. Subsequent GC detection further revealed a concomitant increase in the accumulation of β-elemene. Furthermore, Methylation-Sensitive Amplification Polymorphism (MSAP) analysis revealed that 5-Az altered global DNA methylation patterns. It primarily induced demethylation events. Finally, we explored the nature of these MSAP bands with altered methylation patterns. Gene identification and the effects of 5-Az on terpenoid biosynthesis and methylation not only elucidate the potential role of DNA methylation in secondary metabolism in *C. wenyujin* but also provide novel insights into the molecular mechanisms underlying its geoherbalism. This research opens a new avenue for breeding high-yield and stress-tolerant cultivars.

## Simple Summary

In this study, we conducted the first analysis of cytosine-5 DNA methyltransferase (*C5-MTase*) and DNA demethylase (*dMTase*) genes in *C. wenyujin*. Moreover, we demonstrated that 5-azacytidine (5-Aza)-induced demethylation positively influences the terpenoid biosynthesis pathway and enhances the accumulation of β-elemene, a major sesquiterpene with prominent antitumor activity. This study aims not only to expand our current knowledge of methylation function in *C. wenyujin*, but also to provide a foundation for future research into the relationship between epigenetics and the quality of Dao-di herbs.

## 1. Introduction

DNA methylation, especially 5-methylcytosine (5mC), is a conserved and critical epigenetic modification. In mammals, DNA methylation occurs predominantly in CG sequences [1,2]. In plants, however, it is far more complex, occurring not only in symmetric CG and CHG contexts but also in asymmetric CHH contexts (where H represents A, T, or C) [3]. The precise status of DNA methylation in plants is a dynamic balance, contingent upon the antagonistic processes of methylation and demethylation [4]. DNA methyltransferases, which catalyze the transfer of a methyl group from S-adenosyl-L-methionine to the fifth carbon of a cytosine residue, are classified into four subfamilies based on their conserved domains and functions. Methyltransferase 1 (MET1), which contains two BAH domains, maintains CG methylation during DNA replication [5,6]. Methylation in the CHG context is maintained by Chromomethylase 3 (CMT3) or Chromomethylase 2 (CMT2), which possess a single BAH domain [7,8,9]. Concurrently, CMT2, along with Domain-Rearranged Methylase 2 (DRM2), also participates in CHH methylation [10]. Notably, CMTs are unique to plants and have not been identified in mammals [11]. The DRM subfamily, characterized by UBA domains, is primarily responsible for de novo DNA methylation in all sequences (CG, CHG, and CHH) via the RNA-directed DNA methylation (*RdDM*) pathway [12,13]. In contrast to MET1, CMT2/3, and DRM1/2, DNA methyltransferase homolog 2 (DNMT2)—which lacks a conserved N-terminus—fails to methylate DNA substrates but instead methylates tRNA [14,15]. DNA methylation can be passively lost due to methyltransferase dysfunction or a scarcity of methyl donors. Alternatively, it can be actively removed through a demethylation process initiated by a family of bifunctional DNA glycosylases/lyases. In *Arabidopsis thaliana* (*A. thaliana*), this family includes Repressor of Silencing 1 (ROS1), Demeter (DME), Demeter-like 2 (DML2), and Demeter-like 3 (DML3). These proteins do not directly remove the methyl group from methylcytosine; instead, they excise the entire methylcytosine base from the DNA backbone. The resulting single-nucleotide gap is subsequently filled with an unmethylated cytosine through the base excision repair (BER) pathway [16].

Accumulating evidence suggests that dynamic DNA methylation is closely associated with secondary metabolism in model and crop plants [17]. Overexpression of *AtROS1* in tobacco enhances the demethylation of both promoters and coding regions of genes involved in flavonoid biosynthesis, thereby increasing flavonoid production during salt stress [18]. Similarly, *MdROS1* can decrease the methylation level of anthocyanin-related gene promoters, leading to their increased expression and promoting low-temperature-induced anthocyanin accumulation in apple fruit [19]. Intriguingly, anthocyanin accumulation in apple is also linked to the CHH methylation level of the *MdMYB1* promoter, which is mediated by the *RdDM* pathway [20,21]. Han et al. (2024) found that dynamic DNA methylation levels play an important role in seasonal-dependent secondary metabolism in the new shoots of tea plants [22].

DNA methylation also plays an important role in the secondary metabolic processes in medicinal plants [23]. For instance, in *S. miltiorrhiza* hairy roots, 5-Az treatment significantly enhanced phenolic acid and tanshinone accumulation through epigenetic modulation, while SAM dramatically inhibited these effects [24,25]. A multi-omics analysis of *S. miltiorrhiza* roots at three growth stages revealed that changes in DNA methylation in gene bodies or promoters upregulated tanshinone/phenolic acid biosynthetic genes and downregulated their inhibitors, leading to enhanced compound accumulation [26]. In *D. officinale*, the expression of *DodMTase* and *DoC5-MTase* genes was positively and negatively correlated with water-soluble polysaccharide (WSP) content, respectively, suggesting that DNA methylation may regulate WSP accumulation, although the underlying mechanisms require further investigation [27]. DNA methylation also regulated saponin, chlorogenic acid, iridoid glycoside and flavonoid biosynthesis in *L. japonica*, *E. senticosus*, *R. glutinous,* and *A. annua*, respectively [28,29,30,31]. The secondary metabolites of medicinal plants are not only crucial for new drug research and development but also serve as key criteria for assessing medicinal quality. Given the significant impact of DNA methylation on the accumulation of active ingredients, DNA methyltransferase and demethylase genes have recently been identified in several medicinal species, including *D. officinale*, *S. miltiorrhiza* [32], *C. morifolium* [33], *C. nankingense* [33], *I. rubescens* [34], and *E. senticosus* [29].

As a crucial multifunctional traditional Dao-di geoherb, *Curcuma wenyujin* Y. H. Chen et C. Ling is distributed mainly in Zhejiang Province, China. The essential oil extracted from its tuber is primarily composed of sesquiterpenoids. Although numerous sesquiterpenoids exhibit versatile physiological activities, their levels in *C. wenyujin* are generally low, particularly the pivotal anti-cancer component β-elemene, which fails to meet market demands [35]. Leveraging multi-omics technologies, researchers have made significant efforts to explore the molecular mechanisms underlying the synthesis and accumulation of these bioactive terpenoids. Many key synthase genes have been identified [36,37]. Meanwhile, recent studies showed that methyl jasmonate (MeJA) induced the accumulation of β-elemene in *C. wenyujin* [38]. Further studies found that the CwMYC2-like protein, a bHLH transcription factor, interacted with CwJAZs to respond to JA signaling, thereby regulating β-elemene biosynthesis in *C. wenyujin* [39]. Moreover, CwbHLH27, which activates the transcription of *CwHMGS*, *CwHMGR*, and *CwDXS* by directly binding to the E-box cis-elements within their promoters, interacted with CwJAZ1/17, thereby executing JA signal transduction and regulating sesquiterpenoid biosynthesis in *C. wenyujin* [40]. These findings primarily revealed a complex transcriptional regulatory network governing sesquiterpenoid biosynthesis in *C. wenyujin*. It is well-established that specific ecological conditions are crucial for the formation of geoherbs. These specific secondary metabolites are the outcomes of the adaptation of Dao-di geoherbs to ecological environment changes during long-term evolution. DNA methylation acts as a key mediator in plant responses to environmental factors, thereby influencing the growth of medicinal plants and the biosynthesis of their bioactive compounds [41,42]. However, the impact of epigenetics on these compounds in *C. wenyujin* has not yet been reported. Does DNA methylation also play a regulatory role in the secondary metabolism of *C. wenyujin*? This study aims to investigate this question. Firstly, we performed the identification and analysis of *C5-MTase* and *dMTase* genes in *C. wenyujin*. Furthermore, 5-Az, a DNA methylation inhibitor, can be randomly incorporated into newly synthesized DNA strands, where it irreversibly binds to DNA methyltransferases and inhibits them, leading to a global decrease in genomic methylation. Recent studies have demonstrated that 5-Az effects the accumulation of secondary metabolites by altering the methylation patterns in medicinal plants. Therefore, we treated *C. wenyujin* seedlings with 5-Az to investigate its effects on secondary metabolite production, gene expression, and genomic methylation levels. The MSAP technique was employed to analyze the DNA methylation patterns in *C. wenyujin* before and after 5-Az treatment. This study aims to expand our understanding of DNA methylation function in *C. wenyujin*, while exploring the potential link between epigenetic regulation and its Dao-di authenticity for future research.

## 2. Materials and Methods

### 2.1. Identification and Sequence Analysis of CwC5-MTases and CwdMTases

**Figure 1:**
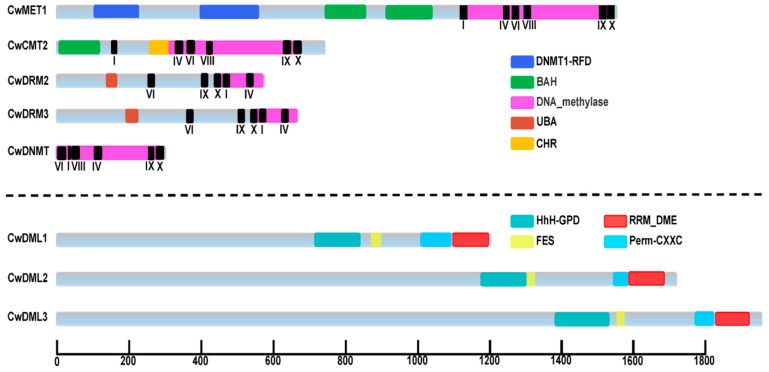
Predicted conserved domains of C5-MTases and dMTases in *C. wenyujin*. DNMT1-RFD (PF12047), cytosine-specific DNA methyltransferase replication foci domain; BAH (PF01426), Bromo-adjacent homology domain; DNA_methylase (PF00145), C-5 cytosine-specific DNA methylase; UBA (PF00627), ubiquitin-associated domain; CHR (SM000165), Ubiquitin associated domain; HhH-GDP (PF00730), helix-hairpin-helix-Gly-Pro-Asp domain; FES (SM000525), 4Fe-4S cluster domain; Perm-CXXC (PF15629), zf-CXXC domain in the Demeter-like proteins and ROS1; RRM-DME (PF15628), RNA-recognition motif domain at the C-terminus of Demeter-like glycosylases; I to X, the conserved motifs in C5-MTase; the numbers on the bottom line indicate the length of proteins.

Hidden Markov models (HMMs) for the DNA methyltransferase domain (PF00145; DNA C5-MTases) and DNA demethylase domains (HhH-GPD: PF00370, RRM_DME: PF15682) were obtained from the Pfam 38.0 database (http://pfam.xfam.org, accessed on 29 November 2025). Due to the lack of genomic information for *C. wenyujin*, these HMM profiles were used as queries to identify candidate *CwC5-MTase* and *CwdMTase* genes within the *C. wenyujin* transcriptome data (SRA accession: PRJNA1161445; https://www.ncbi.nlm.nih.gov/sra/, accessed on 29 November 2025) using the simple HMMER search function in TBtools v1.09 (E-value ≤ 1 × 10^−10^). The coverage and quality of the transcriptome (PRJNA1161445) is detailed in our previous study [43]. Twelve putative *C. wenyujin* C5-MTases (CwMTa1-12) and nine dMTases (CwDML1-9) were confirmed and classified using the SMART version 9 web server (http://smart.embl-heidelberg.de/, accessed on 29 November 2025) and Pfam’s domain search tool. Among the twelve candidate C5-MTases, those lacking the characteristic PF00145 domain (CwMTa5/6/8/9) were excluded. For redundant proteins identified by sequence alignment, only one complete sequence was retained (e.g., CwMTa1/4/11 were kept, whereas CwMTa3/10/12 were removed). With respect to CwdMTases, only the three full-length proteins (CwDML1–3) were retained, while the six partial sequences (CwDML4–9) (despite having PF00730 or PF15628 domains) were excluded. Finally, five CwC5-MTases and three CwdMTases were PCR-cloned and verified by sanger sequencing (Figure 1). Cloning primers are listed in Table S3. Sequence alignments between the CwC5-MTase/CwdMTase proteins and their *A. thaliana* homologs (AtC5-MTase/AtdMTase) were generated using DNAMAN version 6.0 to further characterize the types and distribution of conserved domains and motifs. Ultimately, CwC5-MTase and CwdMTase proteins were classified based on their phylogenetic relationships with their *A. thaliana* counterparts (Figure 1 and Figures S1–S5). The grand average of hydropathy (GRAVY), isoelectric point (pI), and molecular weight (MW) of the CwC5-MTase and CwdMTase proteins were calculated using the ExPASy ProtParam tool (https://web.expasy.org/protparam/, accessed on 29 November 2025). Subcellular localization predictions were performed using Plant-mPLoc version 2.0 (http://www.csbio.sjtu.edu.cn/bioinf/plant-multi/, accessed on 29 November 2025) (Table 1).

### 2.2. Phylogenetic Tree Construction and Conserved Motifs Analysis

**Figure 2:**
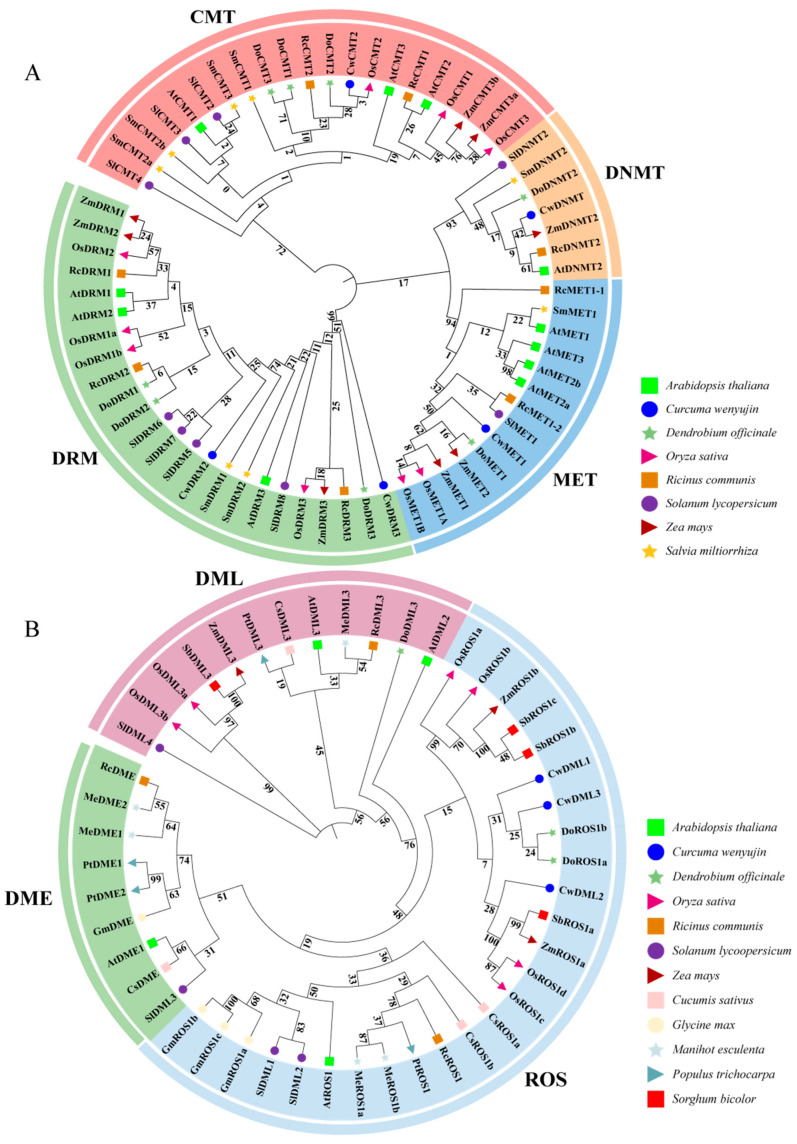
Phylogenetic analysis of the C5-MTase (**A**) and dMTase (**B**) proteins. *At*, *Arabidopsis thaliana*; *Cs*, *Cucumis sativus*; *Cw*, *Curcuma wenyujin*; *Do*, *Dendrobium officinale*; *Gm*, *Glycine max*; *Me*, *Manihot esculenta*; *Os*, *Oryza sativa*; *Pt*, *Populus trichocarpa*; *Rc*, *Ricinus communis*; *Sb*, *Sorghum bicolor*; *Sm*, *Salvia miltiorrhiza*; *Sl*, *Solanum lycopersicum*; *Zm*, *Zea mays*.

To study the evolutionary relationships of C5-MTase and dMTase proteins across diverse plant species, full-length protein sequences from *C. wenyujin*, *A. thaliana*, *C. sativus*, *D. officinale*, *G. max*, *M. esculenta*, *O. sativa*, *P. trichocarpa*, *R. communis*, *S. bicolor*, *S. miltiorrhiza*, *S. lycopersicum*, and *Z. mays* were obtained. The sequences were sourced from Phytozome version 13 (https://phytozome-next.jgi.doe.gov/, accessed on 29 November 2025) or the EBI search database (https://www.ebi.ac.uk/ebisearch/about, accessed on 29 November 2025). The phylogenetic trees were generated using the Maximum Likelihood method with 1000 bootstrap replicates in MEGA 7 software [44] (Figure 2).

**Figure 3:**
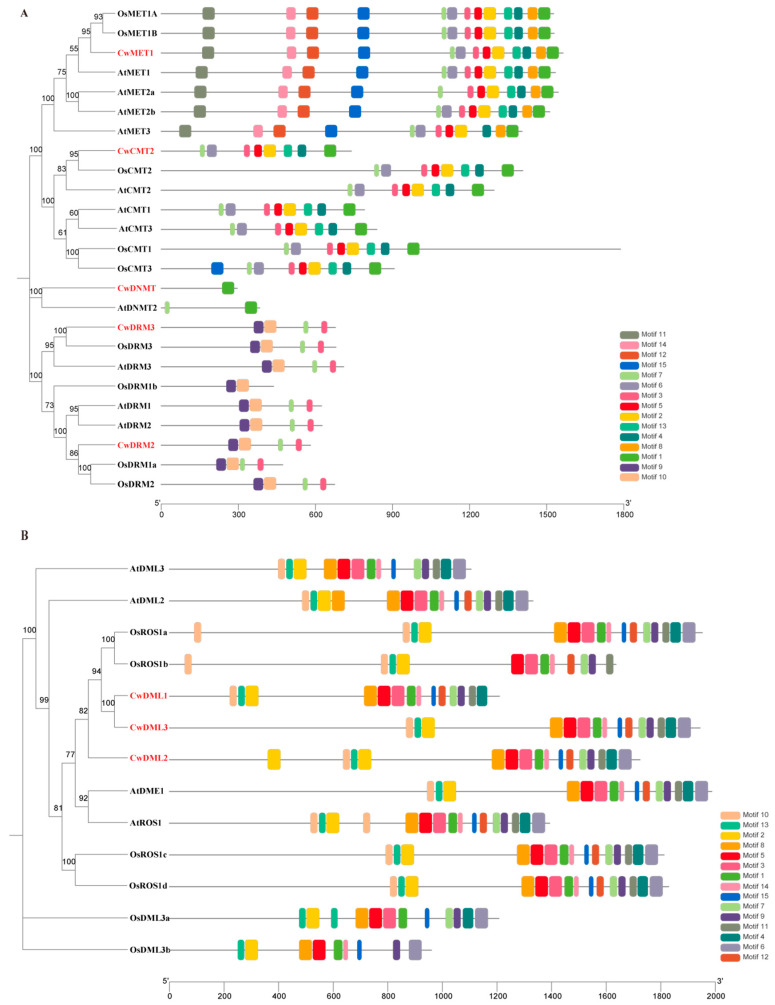
Conserved motifs of C5-MTase (**A**) and dMTase (**B**) proteins from *C. wenyujin*, *A. thaliana,* and *O. sativa*. The numbers on the bottom line indicate the lengths of the proteins.

To further investigate the conservation and divergence of CwC5-MTase and CwdMTase proteins, conserved motifs were analyzed using the online Multiple Expectation Maximization for Motif Elicitation (MEME) software suite (version 5.5.7; https://meme-suite.org/meme/tools/meme, accessed on 29 November 2025). Full-length amino acid sequences of C5-MTase and dMTase proteins from *C. wenyujin*, *A. thaliana*, and *O. sativa* were submitted following the MEME instructions, with parameters set to identify 15 motifs with a width ranging from 6 to 50 amino acids. The resulting motif map was visualized using TBtools v1.09 [45] (Figure 3). The conserved motif sequence information is listed in Figures S6 and S7.

### 2.3. Plant Material and Stress Treatment

In this study, germplasm of *C. wenyujin* (Y.H. Chen and C. Ling) [35] was obtained from Taoshan Town, Ruian, Wenzhou City, Zhejiang Province, China (14 m altitude; 27°47′ N, 120°37′ E). The species identification was performed by Professor Zengxi Guo, who works at the Institute of Food and Drug Control in Zhejiang, China. Following a series of surface sterilization steps, buds excised from seed rhizomes were cultured on Murashige and Skoog (MS) solid medium supplemented with 30 g/L sucrose and 3 mg/L 6-benzylaminopurine (6-BA) to induce shoot clusters. Subsequently, the resulting clustered shoots were divided into individual plantlets and transferred to fresh MS solid medium for approximately two weeks of acclimatization. Once these seedlings reached the four-leaf stage, they were transferred to liquid MS medium supplemented with 50 and 100 μM 5-Az. Leaf samples were collected at 0, 1, 3, and 6 days of treatment, respectively, immediately frozen in liquid nitrogen, and stored in a −80 freezer for subsequent RNA extraction and content analysis. Three biological replicates were prepared for each time point. All plant cultures and treatments were maintained in a growth incubator under a 12 h light/12 h dark photoperiod at 25 °C.

### 2.4. RNA Isolation, RT-qPCR Analysis and Determination of β-Elemene Content

Each collected sample was divided equally into two aliquots, one for total RNA extraction and subsequent gene expression analysis, while the other for the content measurement of β-elemene.

#### 2.4.1. RNA Extraction and Gene Expression Analysis

Total RNA was extracted using the FastPure Plant Total RNA Isolation Kit (RC401-01, Vazyme, Nanjing, China), which is specially designed for polysaccharide and polyphenolic-rich samples. First-strand cDNA was synthesized from 1 μg of total RNA using the PrimeScript™ RT reagent Kit with gDNA Eraser (RR047A, Takara, Beijing, China) according to the manufacturer’s instructions. Quantitative real-time PCR (qRT-PCR) was performed using the LightCycler 96 Real-Time PCR System (Roche Diagnostics, Mannheim, Germany) with ChamQ Universal SYBR qPCR Master Mix (Q711, Vazyme, Nanjing, China). The amplification conditions were as follows: 95 °C for 1 min, immediately followed by 35 cycles of 95 °C for 30 s, 58 °C for 30 s and 72 °C for 30 s, and then 72 °C for 2 min. Data were quantified using the 2^−ΔΔCt^ method based on the Ct values of related genes and the internal control 18S rRNA. Primer sequences used for qRT-PCR are listed in Table S3.

#### 2.4.2. β-Elemene Content Determination

For the determination of β-elemene content, the collected samples were lyophilized using a vacuum freeze-dryer (FreeZone 6 Plus, LABCONCO, Kansas City, MO, USA) for approximately 12 h, after which their dry weight was recorded. The dried samples were then pulverized and extracted overnight with 4 mL of n-hexane in a glass test tube on a horizontal shaker. The next day, approximately 500 μL of the supernatant was collected, filtered through a 0.22 μm organic solvent-compatible membrane into a gas phase vial for analysis. β-elemene content was analyzed using a gas chromatograph (GC-2010 Plus, Shimadzu, Kyoto, Japan) equipped with an Agilent DB-225 capillary column (Agilent Technologies, Santa Clara, CA, USA, 30 m length × 0.25 mm internal diameter × 0.25 μm film thickness). Three biological replicates were analyzed, each with two technical replicates. The detailed GC temperature program, instrumental parameters, and the source of the β-elemene standard used for quantification is described in our previously published work [38].

### 2.5. Methylation-Sensitive Amplified Polymorphism (MSAP) Analysis

Based on the differential sensitivity to site-specific cytosine (5′-CCGG-3′) methylation states of two isoschizomers (*HpaII* and *MspI*), MSAP is a powerful and cost-effective technique for assessing genome-wide DNA cytosine methylation levels, particularly in non-model species lacking a reference genome. In summary, *HpaII* is inhibited by methylation of the external cytosine (the 5′ C in the sequence 5′-C^CGG-3′) in either the hemi-methylated (methylated on one strand) or fully methylated (methylated on both strands) state. However, it cleaves if the internal cytosine (the 3′ C in 5′-CC^GG-3′) is methylated or if the site is unmethylated. *MspI*, in contrast, is inhibited only by methylation of the internal cytosine (5′-CC^GG-3′). It cleaves sites where the external cytosine is methylated (either hemi- or fully methylated) and unmethylated sites [46].

#### 2.5.1. DNA Isolation

The genomic DNA of *C. wenyujin* seedlings was extracted using the improved CTAB method. The quality of the DNA extracts was assessed by 1.0% agarose gel electrophoresis, while the quantity and purity were determined using a NanoDrop spectrophotometer (Thermo Scientific, Vilnius, Lithuania).

#### 2.5.2. MSAP Assay

Genomic DNA (700 ng) was digested with *EcoR I* (20 U)/*Msp I* (10 U) or *EcoR I* (20U)/*Hpa II* (10U) in a total volume of 20 μL at 37 °C for 12 h, respectively, then incubated at 85 °C for 10 min to inactivate the restriction enzymes. Annealed double-stranded adapters were ligated to the digested DNA fragments using T4 ligase (NEB) at 16 °C for 12 h. The ligation products were diluted 5-fold and used as the template for pre-selective amplification. Next, selective amplification was performed using 1/10 dilutions of pre-selective amplification products as the template. The selective amplification procedures were as follows: initial denaturation of 3 min at 94 °C, 12 cycles of 30 s at 94 °C, 30 s at 65 °C (−0.7 °C per cycle), 80 s at 72 °C; followed by 23 cycles of 30 s at 94 °C, 30 s at 55 °C, 80 s at 72 °C, with a final elongation of 10 min at 72 °C. The sequences of the adapters and primers used for pre-selective and selective amplification are provided in Table S4. The final MSAP amplification products were separated on 6% PAGE gels. Gels were stained using silver nitrate and scanned for further data analysis.

#### 2.5.3. Band Scoring

According to their scores of presence (1) or absence (0) for each locus, amplified DNA band patterns of *HpaII*-*EcoRI* and *MspI*-*EcoRI* digestion products were classified into four types: (I) H/M (1/1) represents the unmethylated state at the locus; (II) H/M (1/0) indicates hemi-methylation (methylation on one DNA strand); (III) H/M (0/1) indicates full methylation of the internal C; and (IV) H/M (0/0) is considered an uninformative state, as it could arise from either the absence of the fragment or hypermethylation [47]. The percentage of methylation-sensitive polymorphic bands (reflecting differential methylation states across samples) was calculated using the formula: MSAP Polymorphism (%) = [(II + III + IV)/(I + II + III + IV)] × 100 (Table 2).

## 3. Results

### 3.1. Identification and Classification of CwC5-MTase and CwdMTase Genes in C. wenyujin

Five *C5-MTase* and three *dMTase* genes were identified in *C. wenyujin* (Table 1 and Table S5). Based on their conserved domains and the results of protein sequence alignment from *C. wenyujin* and *A. thaliana*, five *CwC5-MTases* genes were divided into four subfamilies (Figure 1 and Figures S1–S4): *CwMET1*, *CwCMT2*, *CwDRM2/3,* and *CwDNMT*. Three *CwdMTase* genes were named *CwDML1/2/3*, respectively. The open reading frame (ORF) length of the five *CwC5-MTase* genes varies from 891 bp (*CwDNMT*) to 4695 bp (*CwMET1*), encoding proteins ranging from 296 (*CwDNMT*) to 1564 (*CwMET1*) amino acids, with relative molecular weights (MW) between 33.98 and 176.37 kDa. The *CwdMTase* genes, relatively longer, consist of 3630~5835 bp, with the MW ranging from 136.49 kDa (*CwDML1*) to 217.41 kDa (*CwDML3*). This is consistent with the typical length of plant DML proteins, which ranges from 900 to 2000 amino acids. Most of these proteins were predicted to localize to the nucleus, with the exceptions of CwDRM3 and CwDNMT, which were predicted to be in the chloroplast. The grand averages of hydropathicity (GRAVY) values were all negative, ranging from −0.58 (CwDML3) to −0.231 (CwDNMT), indicating that they are hydrophilic proteins. The theoretical isoelectric point (pI) ranges from 4.86 to 8.16. These findings are consistent with results reported for *D. officinale*.

Protein sequence analysis revealed that the C-terminal of CwC5-MTases contains the conserved DNA_methylase domain responsible for catalytic function, while their N-terminal possesses domains characteristic of their respective subfamilies. CwMET1, which belongs to the MET subfamily, harbored two bromo-adjacent homology (BAH) domains at its N-terminus. CwCMT2, a member of the CMT subfamily, had only one BAH domain. The DRM group comprised two members, CwDRM2 and CwDRM3, which featured a ubiquitin-associated (UBA) domain in their N-terminal. The last one is CwDNMT, with no N-terminal conserved domains. Future analysis of the C-terminal region of the CwC5-MTases identified several conserved motifs (I, IV, VI, VIII, IX, and X), arranged in a specific order across different subfamilies (Figure 1 and Figures S1–S4). In addition, the C-terminal of CwDML1/2/3 proteins also contains three conserved regions, region A (Figure 1, Figure S5 orange underline), glycosylase region (Figure 1, Figure S5 purple underline), and region B (Figure 1, Figure S5 red underline), which are discontinuous and interspersed with poorly conserved regions. The glycosylase region includes a HhH-GDP domain and a FES domain, while a Perm-CXXC and an RRM_DME (Figure 1, Figure S5 blue frame) are located in region B.

### 3.2. Phylogenetic and Conserved Motifs Analysis of C5-MTases and dMTases in C. wenyujin and Other Plant Species

To investigate the phylogenetic relationship and evolutionary history of CwC5-MTases and CwdMTases in *C. wenyujin* and other plants species, a phylogenetic tree was constructed using full-length protein sequences of CwC5-MTases and CwdMTases from *C. wenyujin* and 12 other plant species (Tables S1 and S2) using maximum likelihood methods. As shown in Figure 2A, the C5-MTase family was divided into MET, CMT, DRM, and DNMT groups, with CwC5-MTases further classified into corresponding subfamilies, consistent with conserved domains and sequence alignment results. The DNMT subfamily was the smallest, containing only seven members from the eight plant species, whereas the DRM subfamily was the largest in the phylogenetic tree. The phylogenetic analysis indicated a closer evolutionary relationship between the MET and DNMT groups. Additionally, these C5-MTases could be further divided into dicot and monocot groups in each subfamily. CwMET1 and CwDRM3 shared the highest similarity with DoMET1 and DoDRM3, respectively. Meanwhile, CwCMT2 and CwDNMT were clustered into the same subgroups as OsCMT2 and ZmDNMT2, respectively (Figure 2A). This clustering is consistent with the fact that *D*. *officinale*, *O*. *sativa*, *Z*. *mays,* and *C*. *wenyujin* all belong to monocot plants, particularly monocot-medicinal plants for both *C*. *wenyujin* and *D*. *officinale*. Similarly, all the dMTases in these plants were divided into DME, ROS, and DML subgroups, exhibiting significant dicot and monocot differentiation. Among them, CwDMLs shared the highest similarity with DoROS1/2, SbROS1a, ZmROS1a, and OsROS1c/d (Figure 2B).

To further explore the conservation and divergence of CwC5-MTase and CwdMTase proteins, the MEME motif search tool was used to identify conserved motifs in *C. wenyujin*, *A. thaliana*, and *O. sativa*. Fifteen distinct motifs were identified in CwC5-MTase and CwdMTase proteins, respectively. The number of motifs in each CwC5-MTase varied from 1 (CwDNMT) to 13 (CwMET1), with the length ranging from 21 to 50 aa. Among them, motifs 1–5 and 7 were located in all the METs and CMTs in the sequential order 7, 3, 5, 2, 4, 1. Hence, these two subfamily proteins were clustered together in a small branch, while motifs 11, 14, 12, and 8 only existed in the MET subfamily. Motif 3 was present in all C5-MTases except for the DNMT subfamily and OsDRM1b. Motifs 9 and 10 were specific to the DRM subfamily. CwDNMT, the smallest protein of CwC5-MTase, contained only motif 1 (Figure 3A and Figure S6). For the Cwd-MTase, the number of motifs per protein was similar, ranging from 14 (CwDML1) to 16 (CwDML2), with lengths of 19–50 aa. Motifs 1, 2, 5, 9, and 13 were highly conserved in all dMTase proteins. Motifs 8, 14, and 15 were found in 12 of 13 dMTase proteins. Unlike C5-MTase, some motifs, such as motif 10 and 2, appeared twice in the same dMTase protein (Figure 3B and Figure S7). Motifs commonly existing in CwC5-MTases or CwdMTases are probably associated with conserved biological functions, but those specific to a few proteins may be related to gene-specific functions.

### 3.3. Transcript Abundance Analysis of CwC5-MTase and CwdMTase Genes in C. wenyujin

**Figure 4:**
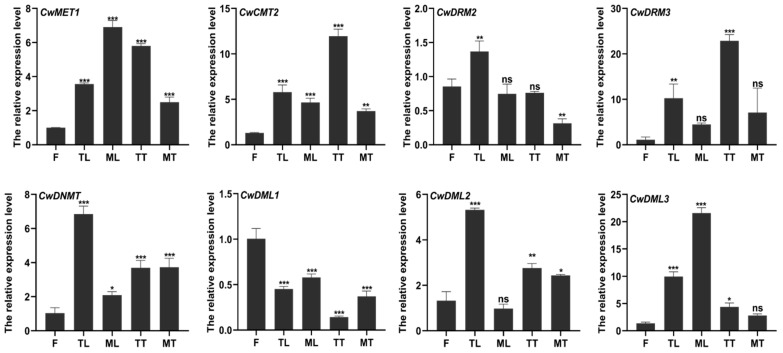
Transcript levels of CwC5-MTase and CwdMTase genes in three C. wenyujin organs. F, flowers; TL, tender leaves; ML, mature leaves; TT, tender tubers; MT, mature tubers. The symbols “ns, *, **, and ***” indicate significant differences at (*p* > 0.05), (*p* < 0.05), (*p* < 0.0003), and (*p* < 0.0002) using Duncan’s multiple range test. Means ± standard deviation (S.D.) are shown. Three biological replicates and three technical replicates were conducted for each data point.

To preliminarily elucidate the biological functions of *CwC5-MTases* and *CwdMTases*, we quantified the transcript abundance of these genes in field-grown *C. wenyujin* across various tissues and developmental stages. The flowers (F) and the tender leaves (TL) were collected from the 3-month-old plants, and the mature leaves (ML), the tender tubers (TT), and the mature tubers (MT) were collected from the 7-month-old plants. As shown in Figure 4, except for *CwDML1*, most genes were highly expressed in leaves and tubers, especially in tender leaves and tender tubers, with lower expression levels in flowers. While *CwCMT2* and *CwDRM3* exhibited peak expression in tender tubers, we ultimately chose to use tissue-cultured sterile seedlings for subsequent experiments for three primary reasons. First, *C. wenyujin* presents challenges for controlled studies due to its long growth cycle and significant phenotypic variability; for instance, plants grown under our laboratory conditions failed to flower. Second, our genes of interest were highly expressed in tender leaves, and a tissue culture system provides a consistent and readily available source of this target tissue. Finally, this sterile system offers practical advantages, including ease of handling, consistent material availability, and minimal risk of experimental contamination.

### 3.4. The Effects of 5-Az on the Expression of Key Genes in the Terpenoid Biosynthesis Pathway and β-Elemene Accumulation

**Figure 5:**
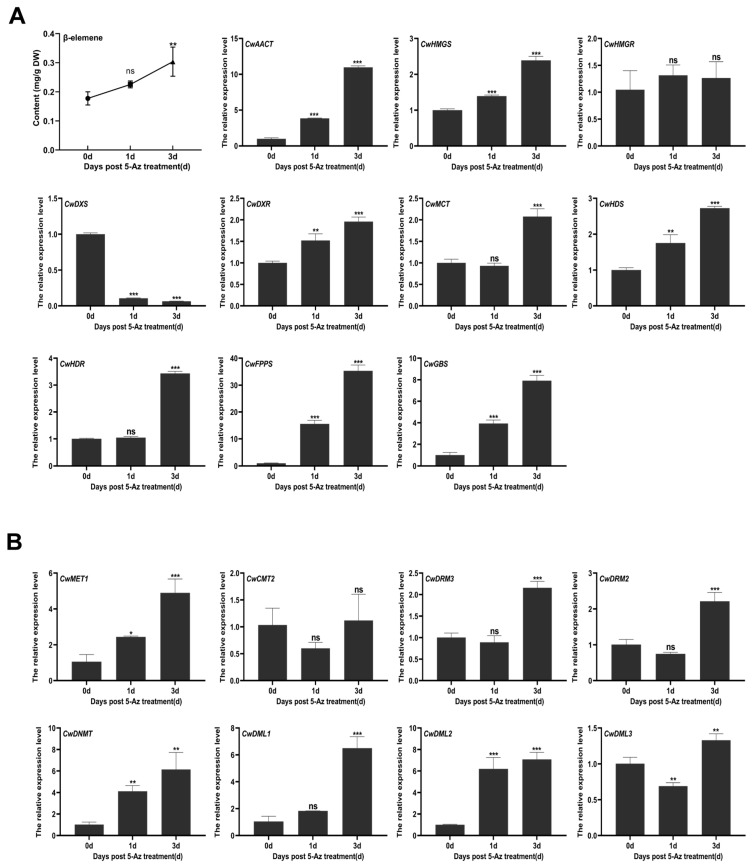
Effects of 100 μM 5-Az on the content of β-elemene and the expression of genes involved in the terpene backbone biosynthesis pathway (**A**) and CwC5-MTase and CwdMTase genes (**B**) in *C. wenyujin*. The symbols “ns, *, **, and ***” indicate the significant difference at (*p* > 0.05), (*p* < 0.05), (*p* < 0.0003), and (*p* < 0.0002) using Duncan’s multiple range test. Means ± standard deviation (S.D.) are shown. Three biological replicates and three technological replicates were conducted for each data point.

In recent studies, treatment with 5-Az was beneficial not only for tanshinone and phenolic acid accumulation in *S. miltiorrhiza* hairy roots [24] but also for the accumulation of other secondary metabolites in different plants [30,48]. To determine whether DNA methylation modification affects β-elemene accumulation and the expression of key enzyme genes in the MVA and MEP pathways, the leaves of *C. wenyujin* seedlings were harvested at different time points after treatment with 5-Az. To determine the optimal 5-Az concentration and treatment duration for our main experiments, a pilot study was conducted. *C. wenyujin* seedlings were treated with 50 μM and 100 μM 5-Az for various time points (e.g., 24, 48, and 72 h), and the effects on plant viability and β-elemene content were assessed to select the final conditions. As shown in Figure S8A, compared with the control group, the content of β-elemene showed no significant change after treatment with 50 μM 5-Az for 1 and 3 days. For the 100 μM 5-Az, we extended the exposure time to 6 days and set up control groups at different time points to exclude potential cytotoxicity of 5-Az on *C. wenyujin* seedlings. As shown in Figure S8B, β-elemene content increased progressively with treatment duration, peaking at 3 days and declining at 6 days, possibly due to reduced 5-Az efficacy or deterioration (e.g., leaf yellowing) in *C. wenyujin* seedlings at 6 days. The β-elemene content of the control groups remained stable at all time points. Consequently, 100 μM 5-Az was applied in follow-up experiments, with sampling at days 0, 1, and 3. As shown in Figure 5A, the relative expression levels of *CwAACT*, *CwHMGS,* and *CwHMGR* in the MVA pathway were measured. *CwAACT* and *CwHMGS* expression increased sharply on day 3, showing approximately 12-fold and 2.5-fold increases compared to the control group, respectively. As for the MEP pathway, with the exception of *CwDXS*, the expression level of most of the key genes was significantly enhanced, including *CwDXR*, *CwMCT*, *CwHDS*, and *CwHDR*. In particular, encoding the key downstream enzyme in the β-elemene biosynthetic pathway, *CwFPPS* showed a remarkable up-regulation on days 1 and 3, reaching 15-fold and 35-fold higher levels than the control group, respectively. Coincidentally, the content of β-elemene showed an upward trend, with an average of 0.28 mg/g (dry weight) in the leaves, compared to 0.17 mg/g (dry weight) in the control group (Figure 5A). In addition, the expression level of *CwGBS*, a germacrene B synthase from *C. wenyujin,* was also significantly upregulated, reaching approximately four and eight times higher than the control groups. It is generally known that germacrene B can be rearranged to generate γ-elemene. The results showed that treatment with 5-Az had a positive influence on the terpenoid biosynthesis pathway. Surprisingly, 5-Az treatment led to increased expression of both *CwC5-MTase* and *CwdMTase* genes, with *CwDML1* and *CwDML2* exhibiting more pronounced up-regulation (Figure 5B). This likely reflects the dynamic antagonism inherent in DNA methylation processes in plants.

### 3.5. The Effects of 5-Az on the DNA Methylation Pattern in C. wenyujin

We investigated whether the stimulating effect of 5-Az in *C. wenyujin* was also accompanied by changes in genomic methylation patterns. Next, we used the MSAP method to assess alterations in DNA methylation patterns of the genomic DNA, and the promoters of *CwFPPS* and *CwGBS* were also investigated using Bisulfite Genomic Sequence (BSP) analysis. To reduce workload and considering that β-elemene content peaks at 3 days of 5-Az treatment, we analyzed the DNA methylation levels of *C. wenyujin* seedlings on day 3 after treatment with 5-Az (3H/3M) or in the control groups (0H/0M, 3HC/3MC). Forty pairs of selective primers were used to analyze the DNA methylation status (Table S4). To distinguish methylation changes induced by 5-Az from those potentially caused by the stress of transferring seedlings from solid to liquid medium, we conducted some preliminary experiments. The control group included not only seedlings at day 0 (before treatment) (0H/0M) but also samples collected at day 3 without 5-Az addition (3HC/3MC) as an additional control. The partial results of PAGE gels in Figure S9 (H17E12, H18E13, H20E11, H19E11) showed that there was no difference in the polymorphism of 3HC/3MC samples compared to 0H/0M, while the methylation pattern of the 5-Az treated samples (3H/3M) for 3 days did change compared to the two control groups. Then we selected the 0-day samples as the control group in subsequent experiments, consistent with the previous samples for β-elemene content determination and gene expression analysis. As shown in Table 2, in all groups, Type I bands were found to be the most frequent fragments, while Types II, III, and IV were detected at lower levels. A total of 803 and 785 fragments were detected in the 5-Az-treated and the control samples, respectively. The total methylation level was calculated according to the sum of Types II, III, and IV bands. Among them, the total number of methylation sites was 99 in the treatment group and 85 in the control group, corresponding to total methylation ratios of 12.33% and 10.83%. Although Type IV bands were detected more frequently than Type II and Type III, it was the changes in Type II (hemi-methylated) and Type III (fully methylated) fragments that were the main source of the altered DNA methylation pattern induced by 5-Az. These findings indicated that 5-Az induced a net demethylation in *C. wenyujin* seedlings, decreasing the total methylation ratio by 1.5%, and that the associated changes primarily stemmed from alterations in the hemi- and fully methylated cytosines (Type II and III).

To explore the dynamic changes in methylation sites under 5-Az treatment, we classified the 14 patterns of bands between the control and treatment that appeared on the MSAP PAGE gels (A1–A3, B1–B5, and C1–C6). As indicated in Table 3, the first pattern with monomorphic bands was classified as Type A, representing no cytosine methylation alteration in the 5′-CCGG-3′ sites. Overall, approximately 87.29% of the CCGG sites remained unchanged under 5-Az treatment. Categories B1 to B5, which represented the cytosine demethylation events, included five categories: II → I, III → I, IV → I, IV → II, and IV → III. The total rate of demethylation events was 9.05%, mainly occurring from hemimethylation to non-methylation (II → I). In contrast, DNA methylation patterns (Type C) comprised four transitions: I → II, I → III, II → IV, and III → IV, while I → IV and II → III were not observed. The total methylation rate was 3.67%. Partial PAGE gel results are shown in Figure S9.

### 3.6. Sequencing Analysis of Differentially Methylated DNA Fragments

To characterize the differentially expressed MSAP bands, a total of 28 fragments were purified, cloned, and sequenced (Table 4). As a reference genome for *C. wenyujin* is not yet available, homologous sequences for only 12 fragments (42.86%) (Figure S9) were identified via BLAST + 2.16.0 searches against the NCBI database (http://blast.ncbi.nlm.nih.gov), while the remaining 16 fragments (57.14%) yielded no significant matches. Most of the twelve fragments were annotated as protein-coding, while the functions of two homologs were undetermined. Among these, five of the twelve fragments were demethylated, and seven were methylated. The length ranged from 120 to 318 bp. According to the annotations of their homologous genes, these fragments may participate in diverse biological processes, including auxin response regulation (IAA17-like protein), abiotic stress responses, plant development, physiology, and secondary metabolism, photosynthesis, and cytokinesis.

## 4. Discussion

In this study, a total of five *CwC5-MTase* and three *CwdMTase* genes were characterized and assigned to their respective subfamilies, including *MET*, *CMT*, *DRM,* and *DNMT2*. Similar to *D. officinale* [27] and *S. miltiorrhiza* [32], only one *MET* gene was found in *C. wenyujin*, while there are four *MET* genes in *A. thaliana* and two in *R. communis* [27], suggesting the loss of *MET* genes during the evolution of *C. wenyujin*. Likewise, we found only *CMT2* and not *CMT3* in *C. wenyujin*, whereas all types of *CMTs* exist in *C. nankingense* [33], *S. miltiorrhiza,* and *D. officinale*. In *Arabidopsis*, although CMT2 can also maintain CHG methylation, this process is mainly performed by CMT3, while CMT2 is also involved in the de novo methylation process mediated by DRM2/3 [3]. Only CMT2 has been identified in *C. wenyujin*, suggesting that the function of CMT2 in genomic methylation may be gradually becoming stronger and has gradually taken over the function of CMT3 during the evolution of *C. wenyujin*. As shown in Figure 2, as in *O. sativa*, *Z. mays,* and *D. officinale*, *CwDML1/2/3* all cluster into the *ROS* subfamily of DNA demethylases, while no *DME* members were identified. Coincidentally, these four plants are all monocots. In Arabidopsis, *DME* is expressed in the central cell of the female gametophyte and plays a critical role in suppressing DNA methylation of maternally imprinted genes in both the central cell and endosperm. However, *C. wenyujin* produces very few seeds, and its cultivation predominantly relies on asexual reproduction. Consequently, the absence of *DME* genes in this species may represent a genuine evolutionary loss rather than a functional redundancy. Although the expansion and contraction of gene families are relatively common in plants [49], the *MET* deletion and the absence of *CMT3*, *DME*, and *DML* in *C. wenyujin* may be due to the identification being based solely on its transcriptome data, which cannot detect non-expressed or lowly expressed genes. It is also possible that other potential homologs were excluded due to low assembly quality of the data. Future studies can supplement these findings with the whole genome sequencing data of *C. wenyujin*.

CwC5-MTases contained six conserved motifs (I, IV, VI, VIII, IX, X) arranged in a characteristic order (Figure 1 and Figures S1–S4), consistent with alignments of bacterial DNA methyltransferases [50,51]. The motif distribution in CwMET1 and CwCMT2 followed a conserved I–IV–VIII–IX–X pattern, whereas CwDRMs displayed a circular permutation, with VI–X preceding I–V, consistent with C5-DRMs in *Arabidopsis* and other plants, suggesting that the rearrangement of the circular permutation occurred before the monocot-eudicot divergence [52]. Two models have been proposed to explain the formation of this circular permutation in plant C5-DRMs [53,54]. As for CwDNMT, this arrangement was very irregular with the order of VI–I–VIII–IV–IX–X (Figure 1 and Figures S1–S4). The high similarity in the types and arrangements of conserved motifs indicates a strong conservation of the sequence and function between *C. wenyujin* and *A. thaliana C5-MTase* genes within the same subfamily. These conserved motifs perform specialized functions in the DNA methylation process [50,51,55]. All three conserved regions found in CwdMTases are essential and sufficient for the catalytic activity of C5-dMTase. A truncated DME variant, DMEΔN677 (lacking 677 aa from the AtDME N-terminus), retains 5mC glycosylase activity, indicating these C-terminal regions are sufficient for function. Similar to other plant C5-dMTases, the FES domain of CwDML1/2/3 contains an iron-sulfur cluster motif with the characteristic spacing Cys-X6-Cys-X2-Cys-X5-Cys (Figure S5, red arrow). Gehring M et al. (2006) [56] and Mok Y G et al. (2010) [57] carried out mutagenesis of all four cysteines and other conserved residues (Figure S5, green stars). These mutant AtDME proteins showed severely impaired or completely abolished 5mC glycosylase activity in vitro [56,57]. The AtROS1 mutants T606L and D611V lost DNA glycosylase activity, while F589 and Y1028 mutations reversed the enzyme’s preference for 5-mC over T. Q607 is essential for flipping out 5-mC, and N608 modulates 5-mC excision in varying sequence contexts [58]. All these residues mentioned above are conserved in CwDML1/2/3 (Figure S5, arrow and stars marked), suggesting that the mechanism of active DNA demethylation in *C. wenyujin* is similar to that in other plants. To elucidate the functions of these genes in *C. wenyujin*, enzymatic activity and subcellular localization validation are recommended in future studies.

For the phylogenetic analysis results (Figure 2), as previously reported [59], each C5-MTase subfamily exhibited distinct monocot–dicot divergence, indicating that the origin of these gene families predates the evolutionary split between monocots and dicots. Notably, the MET and DNMT subfamilies originated from a shared ancestral branch, demonstrating their close phylogenetic relationship. The DNMT subfamily maybe the most “ancient” family among these enzymes [55]. The DRM subfamily is thought to be the most recent to emerge. This is supported by its inverted C-terminal motif arrangement, a feature likely resulting from chromosomal rearrangements. Importantly, this structural arrangement in DRM is consistent across monocots and dicots (Figure S3), suggesting that these rearrangements occurred prior to their divergence.

Next, transcript abundance analysis detected both *CwMTases* and *CwdMTases* in five organs of *C. wenyujin*, but with distinct expression profiles (Figure 4). This variability suggests these genes are involved in the specialized functions and developmental stages of different tissues in *C. wenyujin*. Notably, *CwCMT2*, *CwDRM2/3*, *CwDNMT*, and *CwDML2* were highly expressed in tender leaves (vs. mature leaves), whereas *CwMET1*, *CwDML1*, and *CwDML3* were predominantly expressed in mature leaves. These patterns suggest they may regulate different vegetative growth processes. Tubers, as a primary source of bioactive compounds, actively synthesize and accumulate metabolites during their transition from tender to mature stages, whereas metabolism declines in mature tubers. Gene expression showed that most genes (except *CwDML1* and *CwDNMT*) were more highly expressed in tender tubers than in mature ones, suggesting their potential roles in secondary metabolism regulation. However, *CwDML1* exhibited a unique pattern: it showed the highest expression not only in flowers but also in mature tubers vs. tender tubers. This suggests that *CwDML1* may be critical for floral development, while its function in mature tubers requires further investigation.

Furthermore, significant negative correlations were observed between the β-elemene content (0.17 mg/g vs. 0.28 mg/g) along with the increase in the expression of terpene synthase genes and DNA methylation level (12.33% vs. 10.83%) after treating *C. wenyujin* seedlings with the DNA methylation inhibitor 5-Az for 3 days (Figure 5A, Table 3). Although both methylation and demethylation events occurred following 5-Az treatment, the demethylation sites were the main contributor to changes in the genomic DNA methylation status in *C. wenyujin* seedlings. These findings suggest that the demethylation induced by 5-Az should be beneficial for terpenoid biosynthesis in *C. wenyujin*. As in *E. senticosus,* moderate water deficit reduces DNA methylation at the *EsFPS* promoter, enhancing the synthesis and accumulation of saponins and improving the plant’s drought resistance [60]. However, the regulation of secondary metabolites in medicinal plants is a highly complex process involving multiple layers, including transcription factors, epigenetic and histone modifications, and small RNA-mediated regulation. These mechanisms can influence secondary metabolites individually or collectively by regulating gene expression [61]. For example, the methyl groups added to cytosine directly hinders the binding of NAC transcription factors, including EsJUB1, EsNAC047, EsNAC098, and EsNAC005, to their target promoters and alters the expression of *EsFPS*, *EsSS*, and *EsSE* genes, eventually leading to changes in saponin synthesis in *E. senticosus* [62]. In this study, we did not detect obvious changes in cytosine methylation modifications within the promoters of *CwFPPS* (~600 bp) and *CwGBS* (~2000 bp). This may be due to the fact that DNA methylation enzymes act through broader regulatory networks (TFs: MYB, bHLH, WRKY; epigenetic: histone acetylation) rather than directly binding to key enzyme genes. To our surprise, the expression levels of both *CwC5-MTase* and *CwdMTase* genes were induced by 5-Az (Figure 5B), consistent with concurrent methylation and demethylation events occurring in the genome. In plants, DNA methylation is dynamic, and DNA methylation and active demethylation activities are coordinated. A recent study of the A. thaliana DNA methylome showed that ROS1 activity counteracts RdDM at over 2000 genomic regions. In addition to RdDM mutants, *met1* mutants also showed suppressed *ROS1* gene expression [63]. Methylation-sensitive regulation of demethylase gene expression has also been observed in rice and maize [64,65].

Sequencing and BLAST analysis of differentially methylated DNA fragments annotated to some genes. Among them, IAA17, a member of the Aux/IAA family, is a key regulatory factor in auxin signal transduction. Histone acetylation, a core epigenetic mechanism in plants catalyzed by histone acetyltransferases (HATs), is directly influenced by IAA17 (a phytohormone) through cellular energy metabolism modulation (acetyl-CoA levels), thereby linking auxin signaling to environmental adaptation and developmental processes [66]. Notably, rhizosphere-derived IAA17 suppresses ROS accumulation in plant roots while inducing host DNA methylation-related gene expression, contributing to heavy metal stress resistance [67]. This also aligns with our identification of a metal tolerance protein 2-like factor, suggesting a potential synergy between IAA17-mediated epigenetic regulation and metal detoxification pathways. Currently, MYB proteins, a superfamily of TFs, were confirmed to be involved in the biosynthesis of flavonoids [68], phenolic acids [69], and terpenoids [70] in medicinal plants. In our previous study, the expression levels of *CwTPSs* were increased, as well as the transcription factor *MYBs,* with MeJA treatment [38]. After 5-Az treatment, we indeed identified a methylated MYB gene. Therefore, we propose to investigate the DNA methylation–MYBs–terpene synthase gene regulatory axis in *C. wenyujin* in future studies. These findings showed that transcription factors and epigenetic modifications, such as DNA methylation, histone modification, and small RNA-based mechanisms, may jointly influence secondary metabolites in *C. wenyujin*. The crosslink between them requires further research.

Discrepancies in epigenetic landscapes can often be attributed to different growth conditions. These conditions modulate dynamic changes in the plant metabolome, ultimately facilitating adaptive differentiation among plant varieties. The methylation level of garden ginseng (GG) was higher than that of forest ginseng (FG) [71]. Further studies showed that cold-induced DNA demethylation in American ginseng tender leaves, followed by the high-level expression of *PqFT* and *PqDDS*, ultimately led to maximal ginsenoside accumulation in roots [72]. Beihua and Sijihua are two honeysuckle cultivars. Research has demonstrated that Beihua consistently displays higher levels of total flavonoids compared to Sijihua [73]. A recent multi-omics study revealed a large number of SNP-related differentially methylated cytosines (DMCs) between the two cultivars. These DMCs in the flanking and genic regions of flavonoid biosynthesis genes may lead to their overexpression in Beihua, thereby increasing the flavonoids accumulation [74]. These findings demonstrate that in addition to genetic variations, epigenetic mechanisms—particularly DNA methylation modifications—play an essential role in plant evolution and trait improvement. *C. wenyujin* is renowned for the Chinese class II non-cytotoxic antitumor drug β-elemene, which is a natural sesquiterpene compound with broad-spectrum antitumor effects and a strong ability to inhibit tumor cell migration, with minor side effects [75]. The yield of β-elemene is affected by the raw material variety and origin. *C. wenyujin* from Wenzhou, Zhejiang Province, China, is considered to be the best source on account of its distinctive regional characteristics and high medicinal value. The content of volatile oil, curcumin, and polysaccharides in the rhizomes of *C. wenyujin* cultivated in Wenzhou is higher than that in the rhizomes of *C. wenyujin* grown in Haikou [76]. Although our findings revealed that decreased DNA methylation levels in *C. wenyujin* seedlings accompanied by increased β-elemene accumulation, suggesting that DNA methylation may underlie the geoherbalism of *C. wenyujin*, we have not found direct evidence that DNA methylation is involved in regulating functional genes. Therefore, the link between them remains speculative. In *E. senticosus*, two EsC5-MTase (EsMET1a and EsCMT3b) and two EsdMTase (EsROS1a and EsDME1) were capable of methylation and demethylation of the functional genes in vitro. Simultaneously, in vivo transgenic experiments have confirmed that they can catalyze changes in the methylation status of functional genes, thereby affecting saponin content [29,47]. In the subsequent study, we will conduct similar research in *C. wenyujin*.

Additionally, owing to the absence of a complete reference genome for *C. wenyujin*, we employed MSAP to assess methylation pattern variations, a method favored for its operational simplicity in species with limited genomic resources. However, MSAP is restricted to analyzing CCGG sites and cannot capture non-CpG islands, including CHH contexts, which are critical for understanding regulatory mechanisms in many plants. To address this limitation, we plan to integrate WGBS for comprehensive methylation profiling and HPLC for metabolite quantification in future investigations of *C. wenyujin*.

## 5. Conclusions

In this study, we identified five *CwC5-MTase* genes and three *CwdMTase* genes. Structural and phylogenetic analysis revealed that the five CwC5-MTases were classified into four distinct categories (CwMET, CwCMT, CwDRM, and CwDNMT), whereas the three CwdMTases were assigned to the ROS subfamily. Then, the transcript levels of *CwC5-MTases* and *CwdMTases* were analyzed comprehensively. Further analyses revealed that terpenoid synthase genes were up-regulated, β-elemene content increased, and DNA methylation status was altered in *C. wenyujin* seedlings following 5-Az treatment. While our study provides initial evidence for the potential role of DNA methylation in β-elemene accumulation in *C. wenyujin*, no direct regulatory relationship between DNA methylation and terpenoid synthase genes was identified. Future research should employ in vitro activity assays and in vivo transgenic approaches (overexpression/silencing) to investigate the specific roles of CwMTases and CwdMTases in *C. wenyujin*. The potential connection between DNA methylation and geoherbalism remains speculative, and we propose comparative analysis of methylation patterns across different cultivation regions to address this question.

## Figures and Tables

**Table 1:** Sequence features of *C5-MTase* and *dMTase* genes identified in *C. wenyujin*.

Gene Name	ORF ^a^ (bp)	AA ^b^ (aa)	MW ^c^ (kDa)	GRAVY ^d^ Value	pI ^e^	Predicted Subcellular Localization
*CwMET1*	4695	1564	176.37	−0.469	5.69	Cell membrane, Chloroplast, Cytoplasm, Nucleus.
*CwCMT2*	2223	740	84.08	−0.397	6.34	Nucleus
*CwDRM2*	1746	581	65.13	−0.501	4.98	Nucleus
*CwDRM3*	2037	678	76.76	−0.499	4.86	Chloroplast
*CwDNMT*	891	296	33.98	−0.231	5.43	Chloroplast
*CwDML1*	3630	1209	136.49	−0.564	5.3	Nucleus
*CwDML2*	5175	1724	193.27	−0.57	8.16	Nucleus
*CwDML3*	5835	1944	217.41	−0.58	5.92	Nucleus

^a^ ORF: open reading frame; ^b^ AA: amino acid; ^c^ MW: molecular weight; ^d^ GRAVY: grand average of hydrophobicity; ^e^ pI: theoretical isoelectric point; Subcellular localization was predicted by Plant-mPLoc (http://www.csbio.sjtu.edu.cn/bioinf/plant-multi/, accessed on 29 November 2025).

**Table 2:** MSAP-based cytosine methylation levels under 5-Az treatment and in the control group.

**Type**			**No. of Bands and Ratio**	
*HpaII*	*MspI*		CK	5-Az (3 d)
1	1	I—Non-methylated	704	700
1	0	II—Hemi-methylated	40	32
0	1	III—Fully methylated	33	35
0	0	IV—Hypermethylated	26	18
Total amplified bands			803	785
Total methylated bands			99	85
Fully methylated bands			59	53
Hemi-methylated bands			40	32
Total methylated ratio (%) ^a^			12.33	10.83
Fully methylated ratio (%) ^b^			7.35	6.75
Hemi-methylated ratio (%) ^c^			4.98	4.08
Non-methylated ratio (%) ^d^			87.67	89.17

“1” Presence of band, “0” Absence of band; ^a^: Total methylated ratio (%) = [(II + III + IV)/(I + II + III + IV)] × 100; ^b^: Fully methylated ratio (%) = [(III + IV)/(I + II + III + IV)] × 100; ^c^: Hemi-methylated ratio (%) = [(II)/(I + II + III + IV)] × 100; ^d^: Non-methylated ratio (%) = [(I)/(I + II + III + IV)] × 100.

**Table 3:** Changes in methylation pattern in *C. wenyujin* seedlings under 100 μM 5-Az.

Description of Pattern	Classes	Banding Patterns				No. of Bands	Ratio	Methylation Pattern Alterations
		Control		5-Az (3d)				
		*HspII*	*MspI*	*HspII*	*MspI*			
No change	A1	1	1	1	1	312	87.29%	I → I
	A2	1	0	1	0	24		II → II
	A3	0	1	0	1	21		III → III
Demethylation	B1	1	0	1	1	15	9.05%	II → I
	B2	0	1	1	1	4		III → I
	B3	0	0	1	1	5		IV → I
	B4	0	0	1	0	9		IV → II
	B5	0	0	0	1	4		IV → III
Methylation	C1	1	1	1	0	4	3.67%	I → II
	C2	1	1	0	1	6		I → III
	C3	1	1	0	0	0		I → IV
	C4	1	0	0	1	0		II → III
	C5	1	0	0	0	4		II → IV
	C6	0	1	0	0	1		III → IV

“1”: Presence of band, “0”: Absence of band; Classes: A indicates the same pattern; B indicates a demethylation pattern; and C indicates a methylation pattern.

**Table 4:** BLAST results of polymorphic methylated fragments.

Primer Combinations	Methylation Status	Accession	Description	E-Value
H14E11-1	Methylated	LOC121986379	Auxin-responsive protein IAA17-like	2.8 × 10^−29^
H14E11-2	Methylated	LOC121976864	Polygalacturonase At1g48100-like	2 × 10^−26^
H14E11-3	Methylated	LOC121988566	(S)-2-hydroxy-acidoxidase GLO1-like	1.8 × 10^−21^
H14E12	Methylated	LOC122012221	5-methyltetrahydropteroyltriglutamate--homocysteine methyltransferase 2-like	8.8 × 10^−77^
H14E14-2	Methylated	NC_048505.1	Genome sequence, function unknown	9 × 10^−101^
H17E12	Methylated	LOC121977767	Kinesin-like protein KIN-7C	2.7 × 10^−51^
H18E13	Methylated	OQ909706.1	MYB protein	3 × 10^−9^
H14E13-1	Demethylated	LOC122015080	Metal tolerance protein 2-like	3.5 × 10^−50^
H14E13-2	Demethylated	AP015041.1	Genome sequence, function unknown	2.3 × 10^−3^
H16E20	Demethylated	LOC122045127	Protein SPIRAL1-like3	3.6 × 10^−6^
H17E13	Demethylated	LOC122046078	Flocculation protein FLO11-like	4.1 × 10^−7^
H20E13	Demethylated	LOC121995052	Two-component response regulator ARR10-like	3.2 × 10^−24^

## Data Availability

The transcriptome data of *C. wenyujin* in this study are available in SRA (https://www.ncbi.nlm.nih.gov/sra/, accessed on 29 November 2025) under the accession ID PRJNA1161445 (from SRR30688741 to SRR30688761).

## Supplementary Materials

The following supporting information can be downloaded at: https://www.mdpi.com/article/10.3390/biology14121739/s1, Figure S1: Sequence alignment of MET protein sequences from *C. wenyujin* and *A. thaliana*; Figure S2: Sequence alignment of CMT protein sequences from *C. wenyujin* and *A. thaliana*; Figure S3: Sequence alignment of DRM protein sequences from *C. wenyujin* and *A. thaliana*; Figure S4: Sequence alignment of DNMT protein sequences from *C. wenyujin* and *A. thaliana*; Figure S5: Sequence alignment of DML protein sequences from *C. wenyujin* and *A. thaliana*; Figure S6: Distribution of conserved motifs in CwC5-MTase based on the results of MEME analysis; Figure S7: Distribution of conserved motifs in CwdMTase based on the results of MEME analysis; Figure S8: Effects of 50 μM and 100 μM 5-Az on the contents of β-elemene in *C. wenyujin*; Figure S9: Examples of changing MSAP patterns detected in the treatment samples compared to the control samples; Table S1: Information of C5-MTases genes in the 7 tested species; Table S2: Information of dMTases genes in the 11 tested species; Table S3: Primers used for genes cloning and RT-qPCR analysis in this study; Table S4: Adaptors and primers sequences used for MSAP analysis in this study. Table S5: Information of DNA methyltransferase/demethylase in *C. wenyujin*.
