# Supplementary material for: Identification of DNA Methyltransferase/Demethylase Genes and 5-Azacytidine’s Impact on β-Elemene and Methylation in Curcuma wenyujin
Source: Biology (Basel). 2025 Dec 4;14(12):1739. doi: 10.3390/biology14121739 (PMC12730966; doi:10.3390/biology14121739)
Supplement: Supplementary file 1 [file biology-14-01739-s001.zip › biology-3991885-supplementary/Supplementary Materials.pdf]

Supplementary Materials

Supplementary Figure S1. Sequence alignment of MET protein sequences from *C. wenyujin* and *A. thaliana*.

Supplementary Figure S2. Sequence alignment of CMT protein sequences from *C. wenyujin* and *A. thaliana*.

Supplementary Figure S3. Sequence alignment of DRM protein sequences from *C. wenyujin* and *A. thaliana*.

Supplementary Figure S4. Sequence alignment of DNMT protein sequences from *C. wenyujin* and *A. thaliana*.

Supplementary Figure S5. Sequence alignment of DML protein sequences from *C. wenyujin* and *A. thaliana*.

Supplementary Figure S6. Distribution of conserved motifs in CwC5-MTase based on the results of MEME analysis.

Supplementary Figure S7. Distribution of conserved motifs in CwdMTase based on the results of MEME analysis.

Supplementary Figure S8. Effects of 50 μM and 100 μM 5-Az on the contents of β-elemene in *C. wenyujin*.

Supplementary Figure S9. Examples of changing MSAP patterns detected in the treatment samples compared to the control samples.

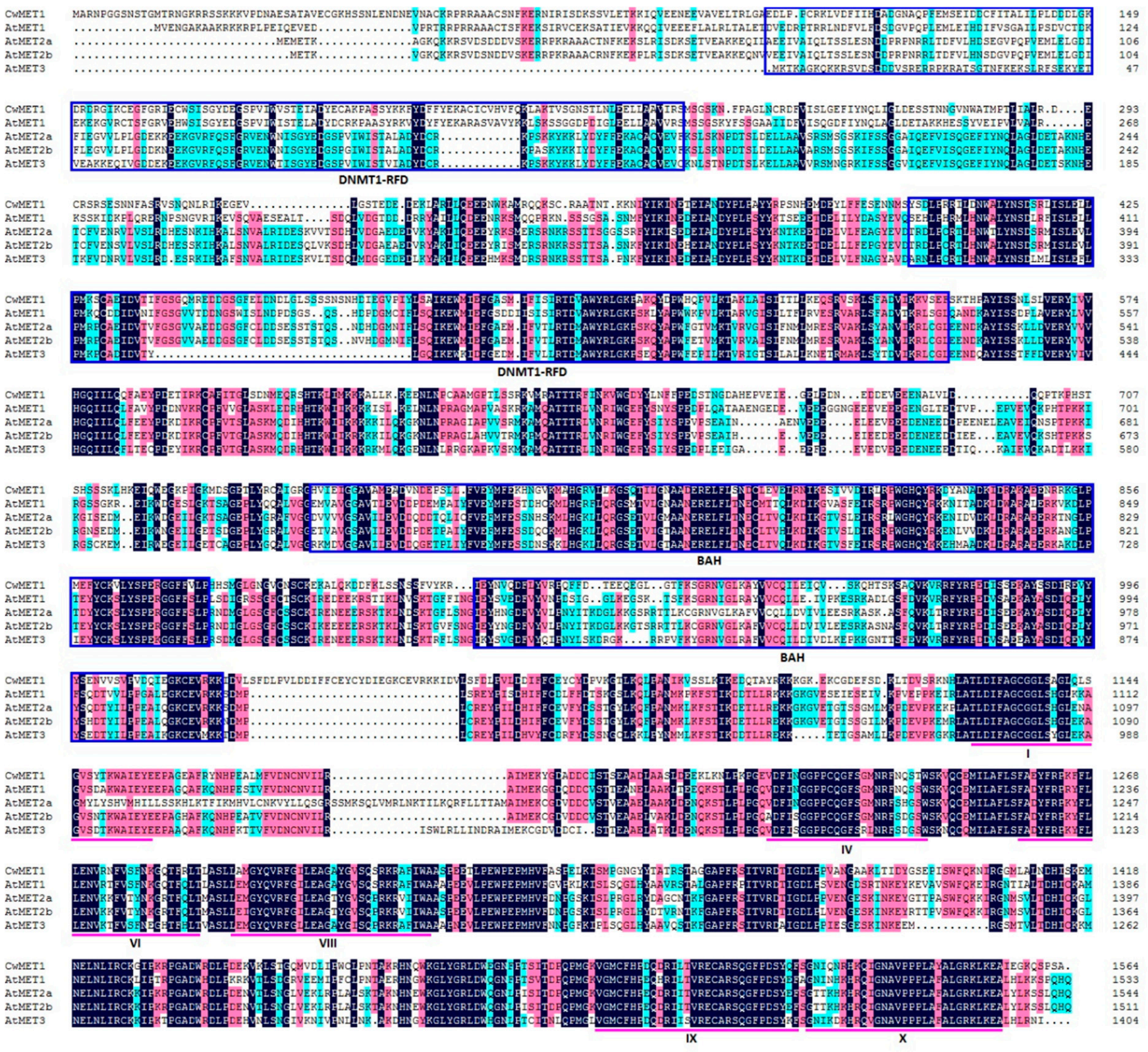

Supplementary Figure S1. Sequence alignment of MET protein sequences from *C. wenyujin* and *A. thaliana.*

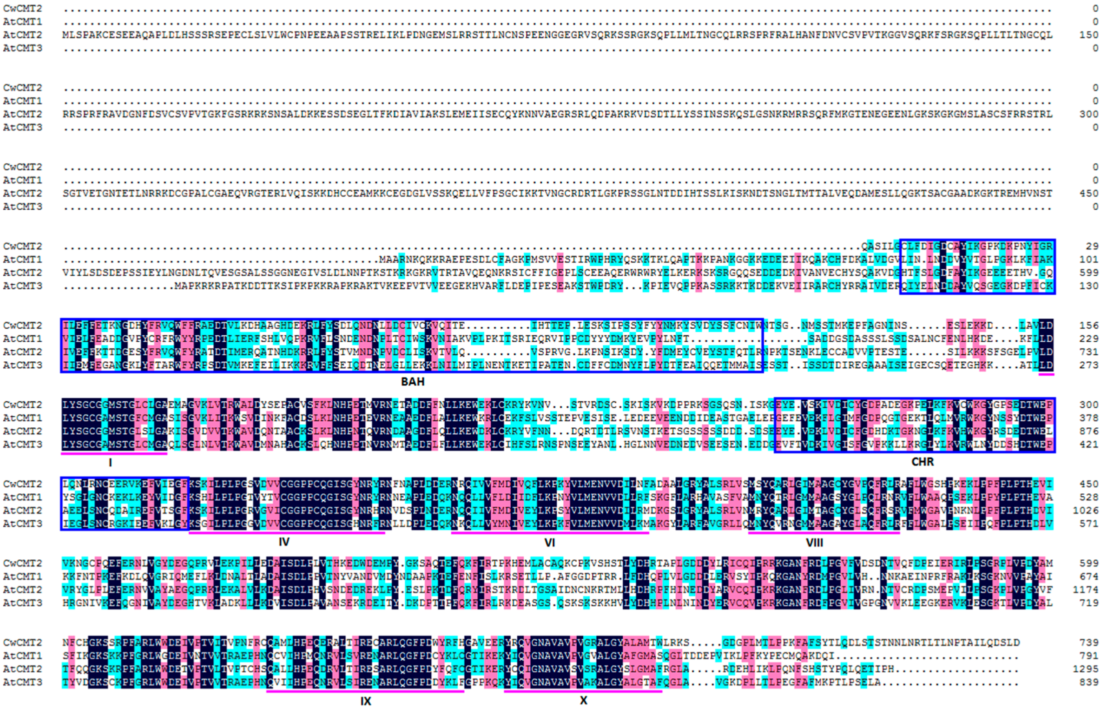

Supplementary Figure S2. Sequence alignment of CMT protein sequences from *C. wenyujin* and *A. thaliana.*

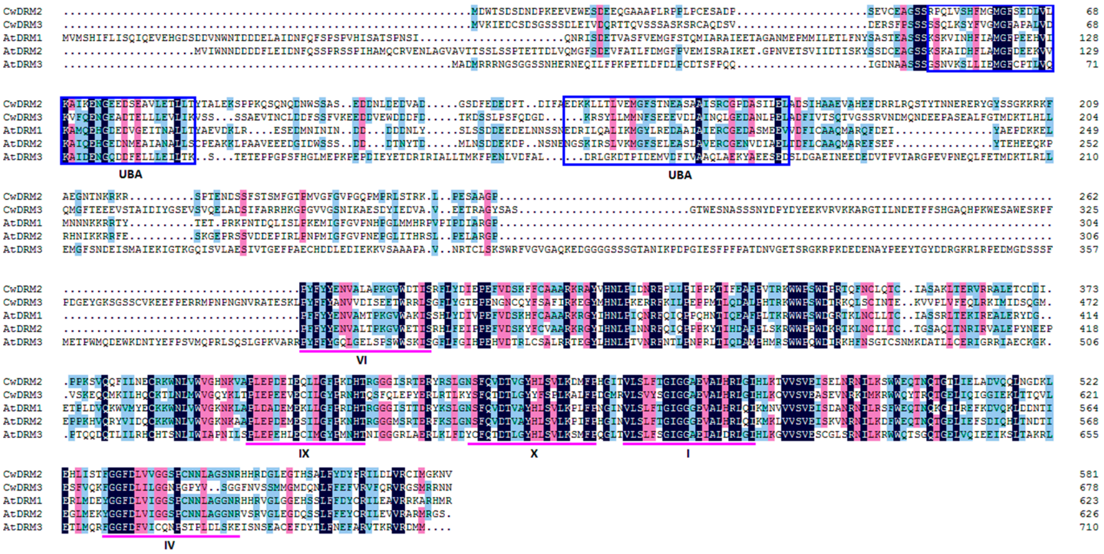

Supplementary Figure S3. Sequence alignment of DRM protein sequences from *C. wenyujin* and *A. thaliana.*

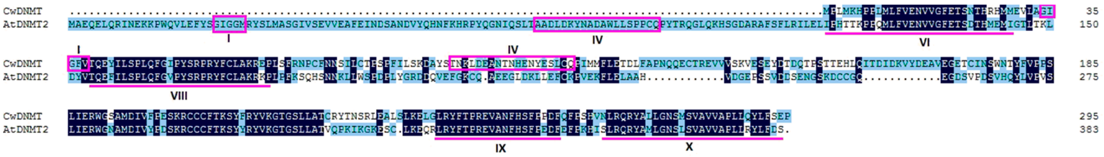

Supplementary Figure S4. Sequence alignment of DNMT protein sequences from *C. wenyujin* and *A. thaliana.*

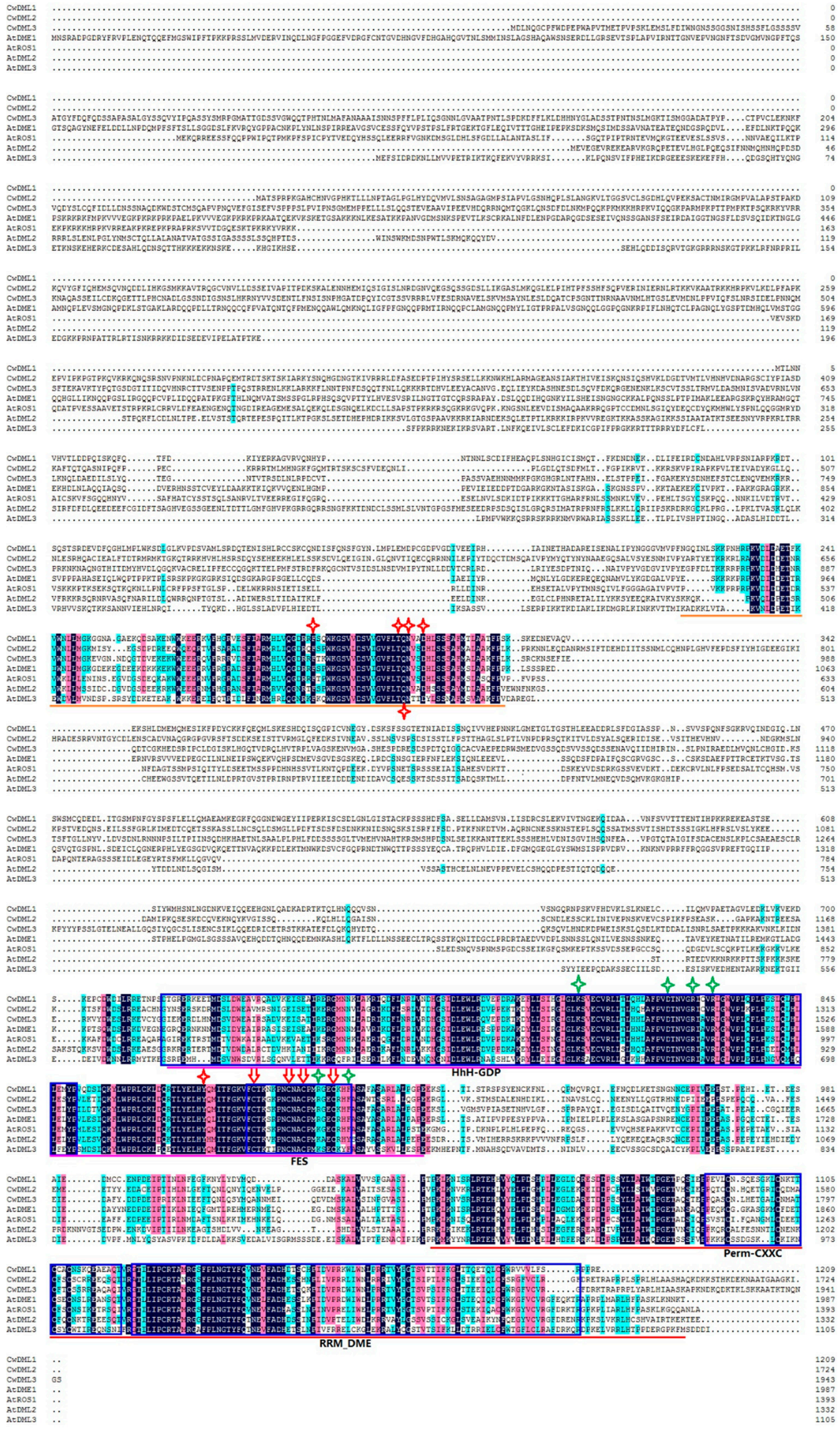

Supplementary Figure S5. Sequence alignment of DML protein sequences from *C. wenyujin* and *A. thaliana.*

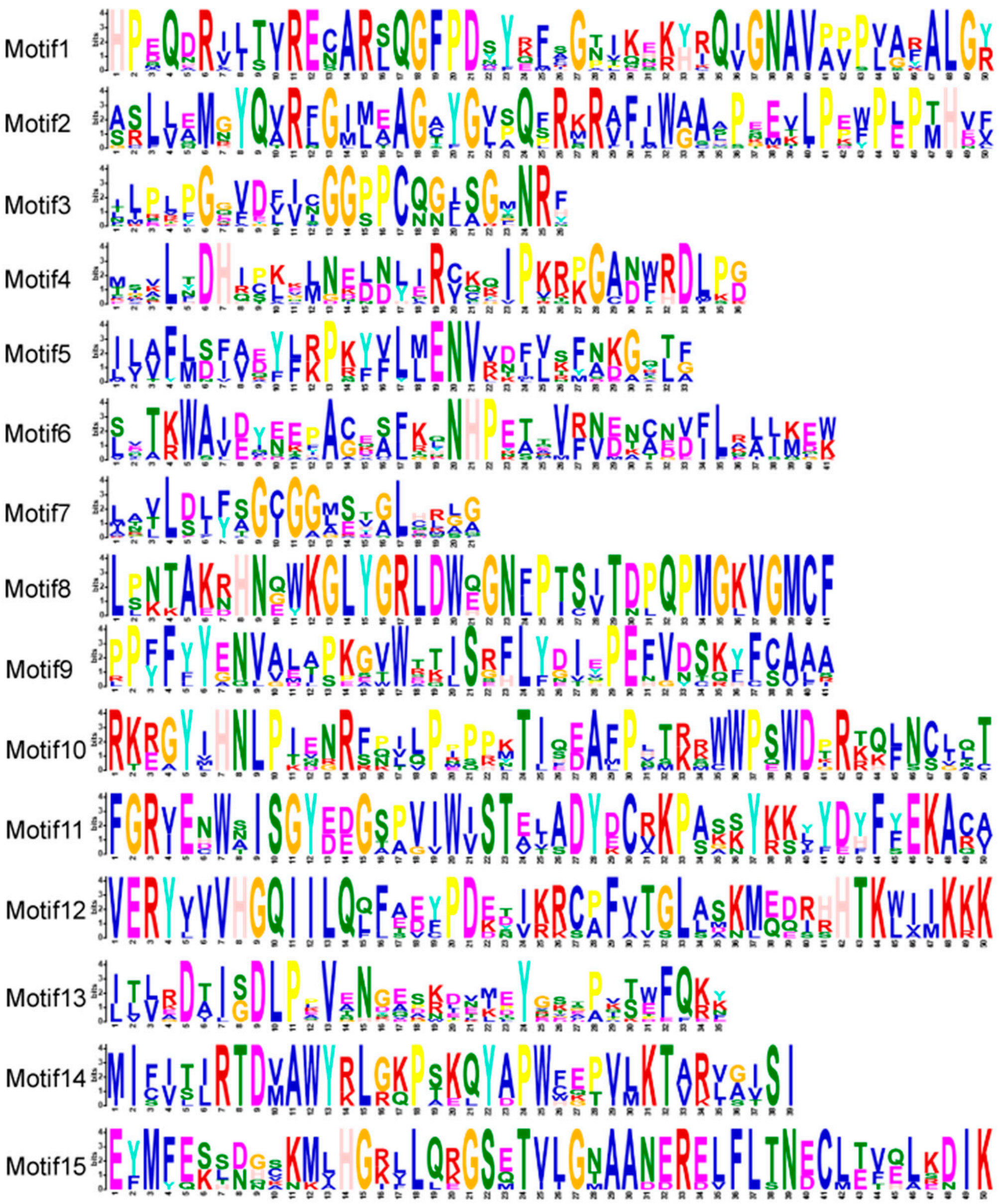

Supplementary Figure S6. Distribution of conserved motifs in CwC5-MTase based on the results of MEME analysis.

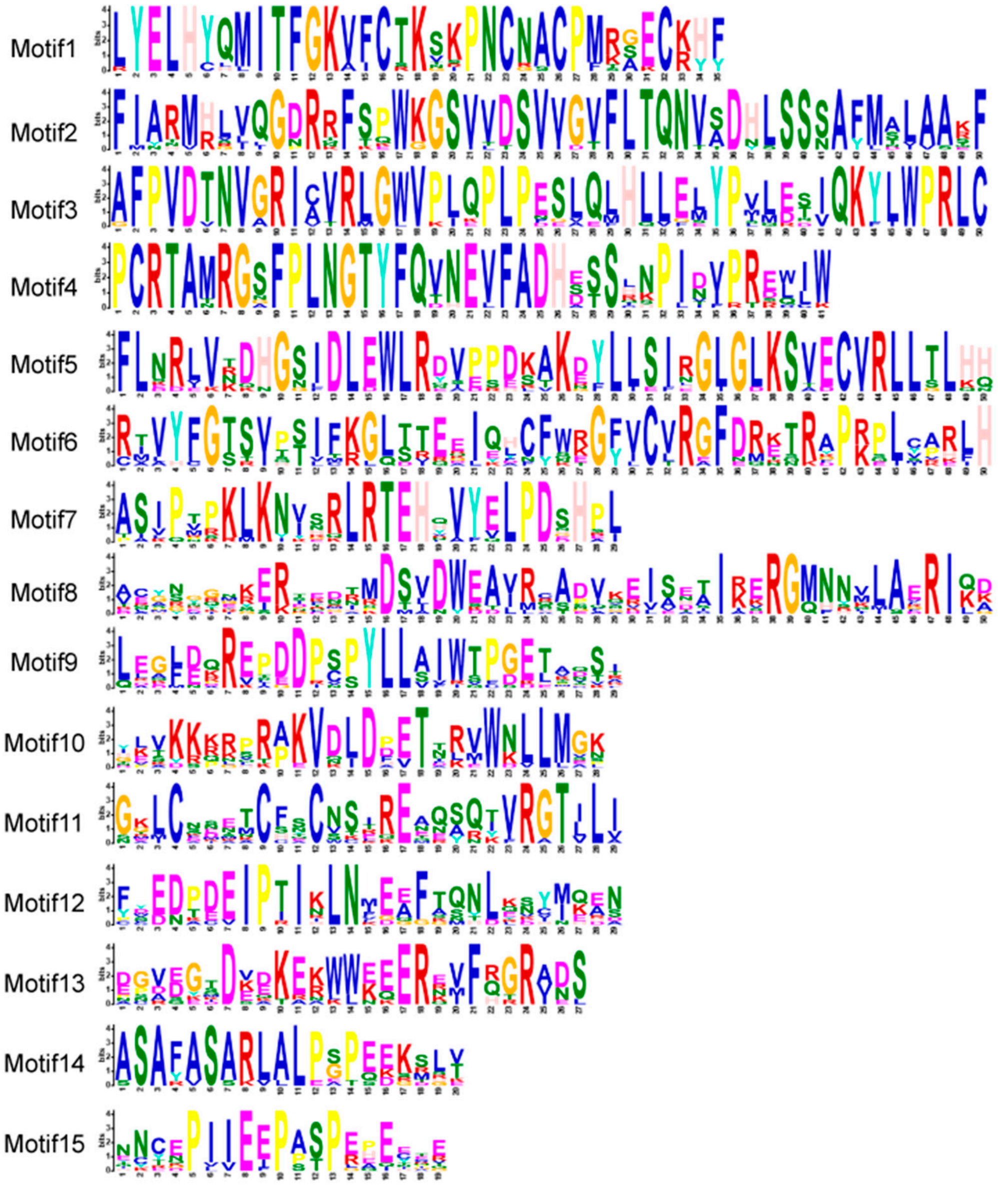

Supplementary Figure S7. Distribution of conserved motifs in CwdMTase based on the results of MEME analysis.

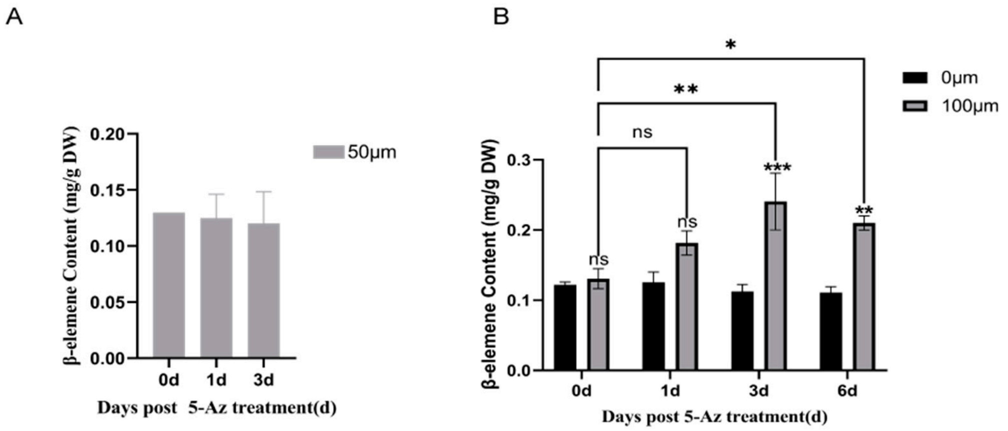

Supplementary Figure S8. Effects of 50 μM and 100 μM 5-Az on the contents of β-elemene in *C. wenyujin*. Effects of 50 μM 5-Az (A) and 100 μM 5-Az (B) on the contents of β-elemene. The symbol "ns, *, **and ***" indicate the significant difference at ($p>0.05$), ($p < 0.05$), ($p<0.0003$), ($p < 0.0002$) using Duncan's multiple range test. Means ± standard deviation (S.D.) are shown. Two biological replicates and three technological replicates were done for each data point.

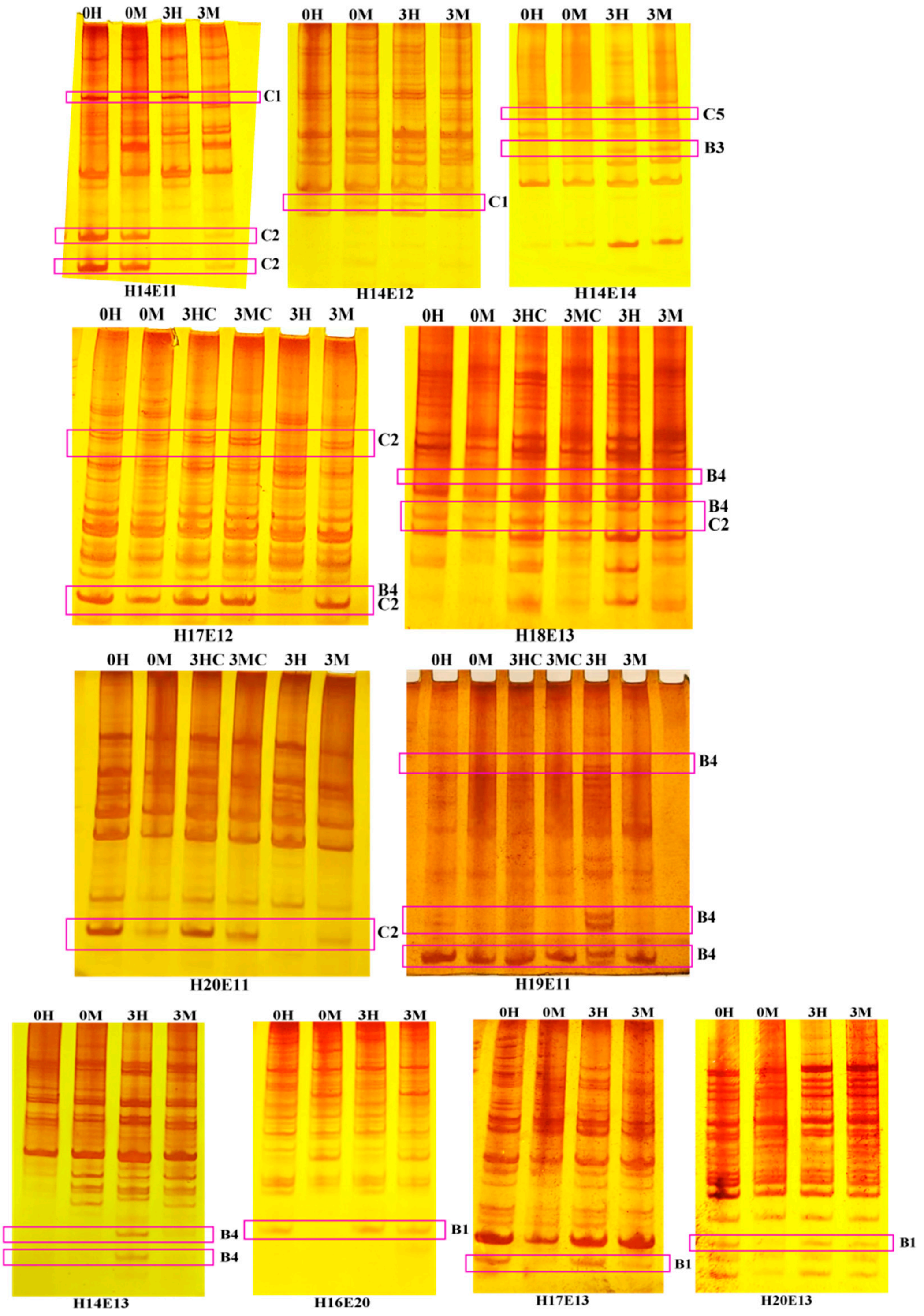

Supplementary Figure S9. Examples of changing MSAP patterns detected in the treatment samples compared to the control samples. 0H, DNA of samples collected on 0 day was digested by EcoRI/HpaII with no 5-Az treatment; 0M, DNA of samples collected on 0 day was digested by EcoRI/MspI with no 5-Az treatment; 3HC, DNA of samples collected on 3 day was digested by EcoRI/HpaII with no 5-Az treatment; 3MC, DNA of samples collected on 3 day was digested by EcoRI/MspI with no 5-Az treatment; 3H, DNA of samples collected on 3 day was digested by EcoRI/HpaII with 100 µM 5-Az treatment; 3M, DNA of samples collected on 3 day was digested by EcoRI/HpaII with 100 µM 5-Az treatment; Typical methylated and demethylated loci were marked by purple frames; B, Demethylation pattern; C, Methylation pattern.
